# Decreased overall mortality rate with Chinese herbal medicine usage in patients with decompensated liver cirrhosis in Taiwan

**DOI:** 10.1186/s12906-020-03010-6

**Published:** 2020-07-14

**Authors:** Fuu-Jen Tsai, Pei-Yuu Yang, Chao-Jung Chen, Ju-Pi Li, Te-Mao Li, Jian-Shiun Chiou, Chi-Fung Cheng, Po-Heng Chuang, Ting-Hsu Lin, Chiu-Chu Liao, Shao-Mei Huang, Bo Ban, Wen-Miin Liang, Ying-Ju Lin

**Affiliations:** 1 School of Chinese Medicine, China Medical University, No. 91, Hsueh-Shih Road, Taichung, Taiwan; 2 Genetic Center, Proteomics Core Laboratory, Department of Medical Research, China Medical University Hospital, Taichung, Taiwan; 3 Asia University, Taichung, Taiwan; 4 Department of Traditional Chinese Medicine, Taipei Medical University Hospital, Taipei, Taiwan; 5 Graduate Institute of Integrated Medicine, China Medical University, Taichung, Taiwan; 6 Rheumatism Research Center, China Medical University Hospital, Taichung, Taiwan; 7 Graduate Institute of Biostatistics, School of Public Health, China Medical University, No. 91, Hsueh-Shih Road, Taichung, Taiwan; 8 Division of Hepato-gastroenterology, Department of Internal Medicine, China Medical University Hospital, Taichung, Taiwan; 9 Chinese Research Center for Behavior Medicine in Growth and Development, 89 Guhuai Road, Jining, Shandong China

**Keywords:** Decompensated liver cirrhosis, Chinese herbal medicine, Overall mortality, Liver fibrosis

## Abstract

**Background:**

Liver cirrhosis is one of the main causes of the morbidity and mortality in liver diseases. Chinese herbal medicine (CHM) has long been used for the clinical treatment of liver diseases. This study was designed to explore the usage frequency and prescription patterns of CHM for patients with decompensated liver cirrhosis and to evaluate the long-term effects of CHM on overall mortality.

**Methods:**

Two thousand four hundred sixty-seven patients with decompensated liver cirrhosis (ICD-9-CM code: 571.2, 571.5, and 571.6) diagnosed between 2000 and 2009 in Taiwan were identified from the registry for catastrophic illness patients. Of these, 149 CHM users and 298 CHM non-users were matched for age, gender, and Charlson comorbidity index score. The chi-squared test, paired Student’s t-test, Cox proportional hazard model, and Kaplan–Meier method were applied for various comparisons between these groups of patients.

**Results:**

CHM-treated patients showed a lower overall mortality risk compared with non-treated patients (Multivariable: *p* < 0.0001; HR: 0.54, 95% CI: 0.42–0.69). The cumulative incidence of overall mortality was lower in the CHM-treated group (stratified log-rank test, *p* = 0.0002). The strongest CHM co-prescription pattern- Yin-Chen-Hao-Tang (YCHT) → Long-Dan-Xie-Gan-Tang (LDXGT) had the highest support, followed by Zhi-Zi (ZZ) → Yin-Chen-Wu-Ling-San (YCWLS) and Bai-Hua-She-She-Cao (BHSSC) → Da-Huang (DaH).

**Conclusion:**

CHM, as adjunct therapy, might decrease the risk of overall mortality in patients with decompensated liver cirrhosis. CHM co-prescription patterns and network analysis showed that comprehensive herbal medicines have a protective role against liver fibrosis. Further studies are required to enhance the knowledge of safety and efficacy of CHM in patients with decompensated liver cirrhosis.

## Background

Liver cirrhosis is a chronic liver disease with liver scarring (liver fibrosis). It is associated with the development of hepatocellular carcinoma, and is also one of the major causes of morbidity and mortality in liver diseases worldwide [1]. In Taiwan, liver cirrhosis is one of the top ten leading causes of death [2]. Liver cirrhosis is characterized by limited liver function with over accumulation of extracellular matrix proteins and is a wound healing reaction to liver injury caused by alcoholism, hepatitis B and/or hepatitis C virus infections, and nonalcoholic steatohepatitis [3].

Clinically, liver cirrhosis may either be compensated or decompensated [4]. Compensated liver cirrhosis is characterized by poor but still relatively preserved liver function, while decompensated liver cirrhosis is considered as the extensive and progressive loss of liver function. Ascites is the most frequent symptom, followed by gastrointestinal bleeding, microbial infection, and hepatic encephalopathy. Following appearance of these characteristics, this disease usually progresses rapidly towards death or requires liver transplantation. Management of liver cirrhosis is often focused on preventing liver-related morbidity and mortality and improving the quality of life. Thus, it is urgent to develop and provide effective therapeutic strategies for these patients.

Traditional Chinese medicine (TCM) is popular as adjunct treatment and has the potential to reduce the morbidity and mortality in liver diseases [5]. Chinese herbal medicine (CHM) belongs to TCM and has been applied as adjunct therapy for several diseases to improve disease-related complications and mortality in Taiwan [6–15]. Given that liver cirrhosis is one of the top ten leading causes of death in Taiwan, this study focused on patients with decompensated liver cirrhosis from the registry for catastrophic illness patients in Taiwan. This study was designed to explore the long-term effects of CHM on these patients with decompensated liver cirrhosis.

## Methods

### Data resource

For this study, data was retrieved from the National Health Insurance Research database (NHIRD; from 1996 to 2012), (http://nhird.nhri.org.tw/) of the National Health Insurance (NHI) program in Taiwan (https://www.nhi.gov.tw/english/). This database is managed by the National Health Research Institute (NHRI) and consists of data from a large longitudinal and retrospective cohort of 1 million people randomly sampled from the total population of 24 million. Information contains medical records including age, sex, symptoms, diagnosis of disease, drug prescription, procedures, record of clinical visits and hospitalizations, inpatient orders, ambulatory care, and sociodemographic factors. The medical records collected from this database were anonymized. The study protocol was approved by the Institutional Review Board of China Medical University Hospital.

### Study population

**Fig. 1:**
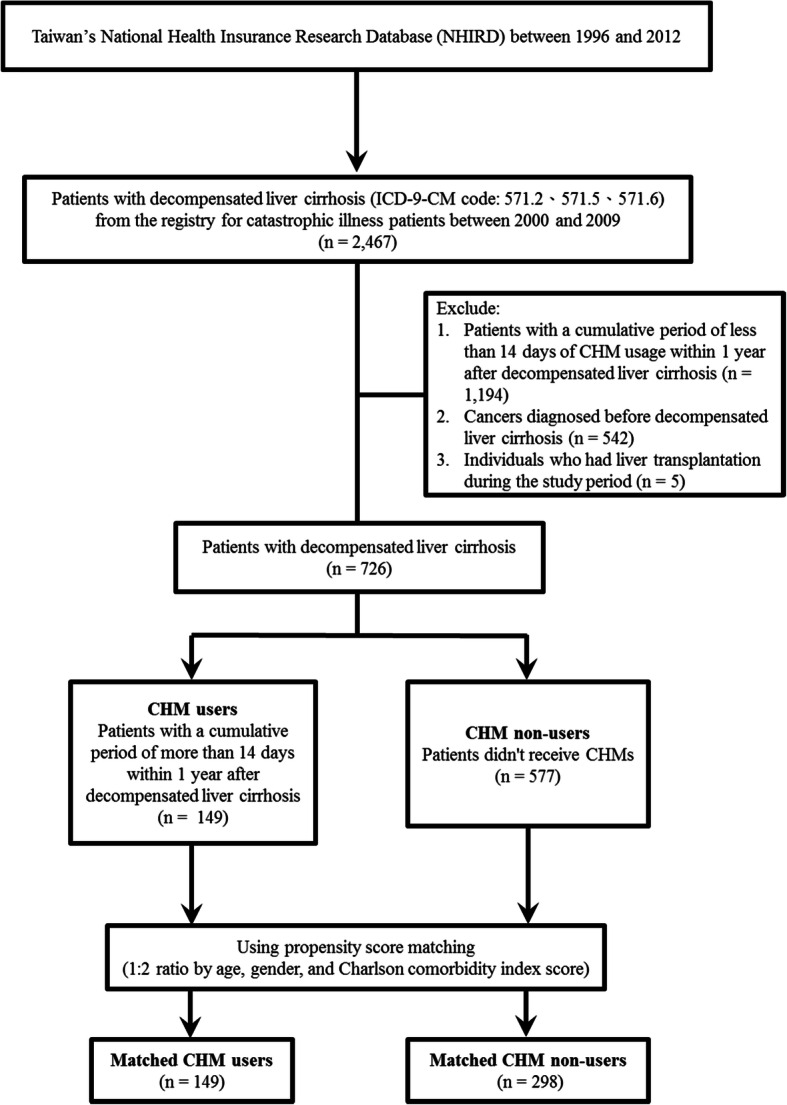
A flow diagram presenting the study participants enrollment

The International Classification of Diseases, 9th Revision, Clinical Modification (ICD-9-CM) was used for the identification of the study population. We conducted a longitudinal and retrospective cohort study with individuals newly diagnosed with decompensated liver cirrhosis (ICD-9-CM code: 571.2, 571.5, and 571.6) between 2000 and 2009 from the registry for catastrophic illness patients (Fig. 1). The first record was considered as the date of diagnosis. The exclusion criteria for this study were (1) patients with less than 14 cumulative days of CHM use within 1 year after diagnosis of decompensated liver cirrhosis; (2) cancer diagnosed before decompensated liver cirrhosis; and (3) individuals who had undergone liver transplantation during the study period. Patients were defined as the CHM users who had more than 14 cumulative days of CHM treatment within the first year of diagnosis of decompensated liver cirrhosis (*n* = 149, Fig. 1). Patients were defined as the CHM non-users who did not have any recorded use of CHM (*n* = 577).

There are two forms of CHM - herbal formula and single herb. The formula contains a combination of at least two single herbs prescribed by experienced TCM doctors according to ancient medical books (Table S1). Most single herbs are derived from plants (Table S1). Only Hai-Piao-Xiao (HPX), which is a single herb extracted from bones of *Sepia officinalis* (Cuttlefish Bone) was factory-made by Good Manufacturing Practice certificated pharmaceutical companies within Taiwan (Supplementary file from Chuang Song Zong Pharmaceutical Co. Ltd. (http://www.csz.com.tw/)). For CHM users, their prescription frequency, usage frequency, person-years, average drug dose (gram/per day), and average duration of prescription (days) were considered (from the index date to the end of the study) according to each herbal formula and single herb (Table S1). The CHM products were produced by the various pharmaceutical manufacturers in Taiwan (Chuang Song Zong Pharmaceutical Co. Ltd. (http://www.csz.com.tw/), Shang Chang Pharmaceutical Co. Ltd. (http://www.herb.com.tw/about_en.php), Sun Ten Pharmaceutical Co. Ltd. (http://www.sunten.com.tw/), Kaiser Pharmaceutical Co. Ltd. (http://www.kpc.com/), and KO DA Pharmaceutical Co. Ltd. (http://www.koda.com.tw/)).

To avoid potential confounding factors, both groups were further matched with propensity score (1:2 matching for age, gender, and Charlson comorbidity index score) (Table 1). There were 149 matched CHM users and 298 CHM non-users. The end of 14 cumulative days of CHM use within 1 year was chosen as the index date. During this study period, these CHM users also used CHM products (Table S5). The study endpoint was a composite of overall mortality, the date of withdrawal from the NHI program, or the date of termination of follow-up (December 31, 2012). Characteristics of CHM and non-CHM using patients with decompensated liver cirrhosis in Taiwan are shown in Table 1. These included age, gender, Charlson comorbidity index score, interferon therapy, anti-viral therapy, income, and urbanization level. Comorbidities were identified before diagnosis of decompensated liver cirrhosis [16, 17]. Income was divided into three subgroups (Table 1; <NT20,000, NT20,000-NT30,000, and ≧NT30,000). Urbanization levels in Taiwan was divided into three subgroups according to the Taiwan National Health Research Institute publications, with level 1 referring to the lowest level of urbanization and level 3 referring to the highest level.

**Table 1:** Demographic characteristics of decompensated liver cirrhosis patients according to CHM usage in Taiwan

Characteristics	Total subjects		***p***-value	Matched subjects		***p***-value
	CHM users	Non-CHM users		CHM users	Non-CHM users	
	(***N*** = 149)	(***N*** = 577)		(***N*** = 149)	(***N*** = 298)	
	N (%)	N (%)		N (%)	N (%)	
**Age (Mean ± SD)**	53.40 ± 12.54	54.82 ± 14.17	0.265	53.40 ± 12.54	54.47 ± 13.3	0.249
**Gender**			***0.036***			1.000
Male	106 (71.14%)	457 (79.2%)		106 (71.14%)	212 (71.14%)	
Female	43 (28.86%)	120 (20.8%)		43 (28.86%)	86 (28.86%)	
**Charlson comorbidity index score (Mean ± SD)**	4.34 ± 2.12	4.65 ± 2.41	0.151	4.34 ± 2.12	4.21 ± 2.39	0.389
**Interferon therapy**	2 (1.34%)	3 (0.52%)	0.279	2 (1.34%)	3 (1.01%)	0.750
**Anti-virus therapy**	2 (1.34%)	3 (0.52%)	0.279	2 (1.34%)	3 (1.01%)	0.750
**Income**			***0.006***			***0.017***
< NT20,000	81 (54.36%)	387 (67.07%)		81 (54.36%)	198 (66.44%)	
NT20,000-NT30,000	40 (26.85%)	128 (22.18%)		40 (26.85%)	69 (23.15%)	
≧NT30,000	28 (18.79%)	62 (10.75%)		28 (18.79%)	31 (10.4%)	
**Urbanization level**			0.553			0.538
1	77 (55.40%)	296 (53.72%)		77 (55.4%)	152 (53.15%)	
2	40 (28.78%)	146 (26.50%)		40 (28.78%)	76 (26.57%)	
3	22 (15.83%)	109 (19.78%)		22 (15.83%)	58 (20.28%)	

*p*-values for gender, interferon therapy, anti-virus therapy, income, and urbanization level were calculated with chi-square test

For matched subjects, *p*-values for age and Charlson comorbidity index score were calculated using paired Student’s t-test

*CHM* Chinese herbal medicine; *N* number

These comorbidities were identified before decompensated liver cirrhosis

Propensity score matching was performed for CHM and non-CHM users in 1:2 ratio for age, sex, and Charlson comorbidity index score

Urbanization level: 1 indicates the lowest level of urbanization and 3 is the highest level

### Statistical analysis

**Fig. 2:**
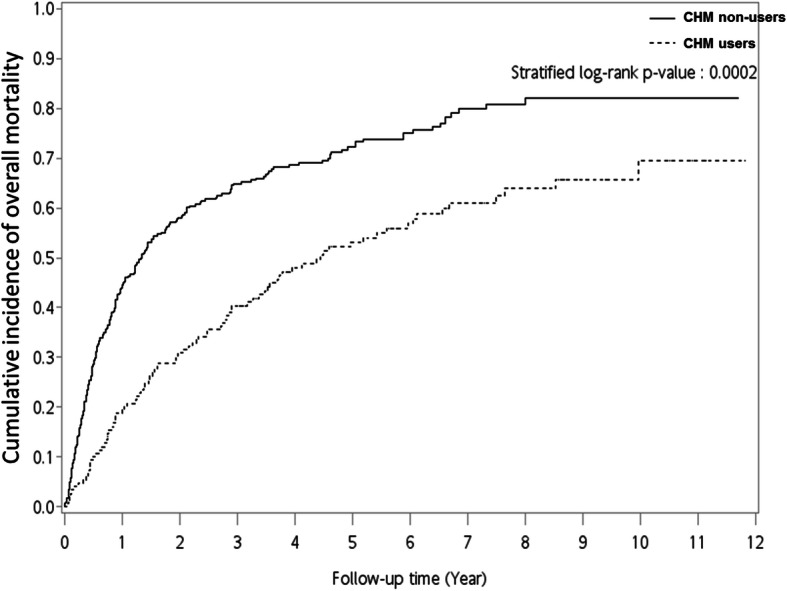
The cumulative incidence of overall mortality in decompensated liver cirrhosis patients based on usage of Chinese herbal medicine (CHM)

**Fig. 3:**
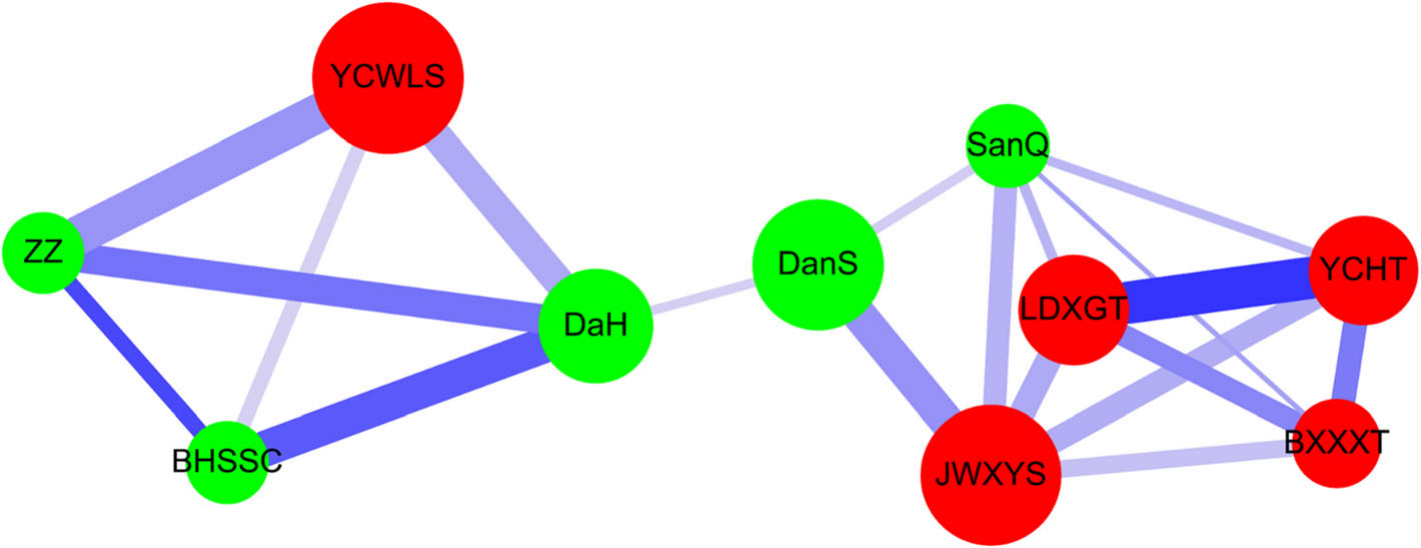
The CHM network in patients with decompensated liver cirrhosis. The lines connecting CHMs represent the support value, where thicker lines represent higher support values, and darker lines represent higher lift values. The thicker and darker the connecting line, the more important the connection between CHMs. The size of the circle represents the frequency of prescription of individual CHMs i.e., larger circles represent higher prescription frequencies. Red circles represent herbal formulas and green circles represent single herbs

Categorical data are expressed as absolute number (percentage) and were compared using Chi-squared tests (Table 1). For un-matched and matched subjects, *p*-values for age and Charlson comorbidity index score were calculated using regular Student’s t-test and paired Student’s t-test (Table 1 and Table S3). Cox proportional hazard models with robust sandwich variance estimator were used to evaluate the hazard ratio (HR) of the risk of overall mortality with adjusted factors (Table 2 and Table S4). Regular Cox proportional hazard models were used to evaluate the hazard ratio (HR) of overall mortality with adjusted factors (Table S2). Adjusted factors included age, gender, Charlson comorbidity index score, CHM use, and income. The Kaplan-Meier method and the stratified log-rank test were used to assess the 12-year cumulative incidence of overall mortality (Fig. 2 and Fig. S3). The Kaplan-Meier method and the log-rank test were used to assess the 12-year cumulative incidence of overall mortality (Fig. S1). Parameters for counting the cumulative incidence of overall mortality are shown for CHM and CHM non-users (Table S7 and Table S8). Interval (Lower; Upper) refers to the follow-up time; for example: lower = 0; upper = 1 means the interval between 0 and 1 year. Effective sample size (n) refers to the total sample number in the CHM or CHM non-users. n = N-1/2 (NC); where number of censored (NC) means the number of withdrawal or loss of follow-up during the interval in the two groups. Conditional probability of failure was calculated as (q) = NF/n. Survival was calculated as (p) = Π *p* = Π(1-q). Overall mortality was calculated as 1-Survival. Co-prescription pairs of CHM products were shown by using association rules [18] (the “arules_1.6” package of R software (version 3.4.3); Table 3). Cytoscape network analysis (http://manual.cytoscape.org/en/stable/Network_Analyzer.html) was used to investigate the CHM network (Fig. 3). All *p*-values less than 0.05 were considered statistically significant. Statistical analyses were performed using SAS software (version 9.4; SAS Institute, Cary, NC, USA).

**Table 2:** Hazard ratios (95% CI) for overall mortality of decompensated liver cirrhosis patients

	Number of death (***n*** = 313)	Total (***n*** = 447)	Crude			Multivariable		
	N (%)	N	Hazard ratio	95% CI	***p***-value	Hazard ratio	95% CI	***p***-value
**CHM use (vs. non-CHM use)**								
No	225 (75.50%)	298	Ref.	Ref.	Ref.	Ref.	Ref.	Ref.
Yes	88 (59.06%)	149	0.54	(0.43–0.68)	***< 0.0001***	0.54	(0.42–0.69)	***< 0.0001***
**Age (per year)**	ND	ND	1.02	(1.01–1.03)	***0.0007***	1.01	(1.00–1.02)	***0.0119***
**Gender**								
Male	216 (67.92%)	318	Ref.	Ref.	Ref.	Ref.	Ref.	Ref.
Female	97 (75.19%)	129	1.32	(1.05–1.68)	***0.0196***	1.03	(0.80–1.33)	0.8018
**Charlson comorbidity index score (per score)**	ND	ND	1.08	(1.03–1.13)	***0.0023***	1.07	(1.01–1.13)	***0.0210***
**Income**								
< NT20,000	220 (78.85%)	279	Ref.	Ref.	Ref.	Ref.	Ref.	Ref.
NT20,000-NT30,000	58 (53.21%)	109	0.53	(0.39–0.72)	***< 0.0001***	0.54	(0.39–0.74)	***0.0001***
≧NT30,000	35 (59.32%)	59	0.58	(0.41–0.82)	***0.0024***	0.64	(0.44–0.93)	***0.0203***

*CHM* Chinese herbal medicine; *HR* hazard ratio; *95% CI* 95% confidence interval; *Ref* reference; *ND* not determined

Adjusted factors included age, gender, Charlson comorbidity index score, CHM use, and income

Cox proportional hazard models with robust sandwich variance estimator were applied in this analysis

**Table 3:** Ten most commonly used pairs of CHM products for decompensated liver cirrhosis patients in Taiwan

CHM products (LHS, X)	Chinese name		CHM products (RHS, Y)	Chinese name	Frequency of prescriptions of X and Y products	Support (X) (%)	Confidence (X → Y) (%)	Lift
Yin-Chen-Hao-Tang (YCHT)	茵陳蒿湯	→	Long-Dan-Xie-Gan-Tang (LDXGT)	龍膽瀉肝湯	144	3.81	43.24	4.83
Zhi-Zi (ZZ)	梔子	→	Yin-Chen-Wu-Ling-San (YCWLS)	茵陳五苓散	135	3.57	68.53	4.80
Bai-Hua-She-She-Cao (BHSSC)	白花蛇舌草	→	Da-Huang (DaH)	大黃	128	3.38	63.05	6.65
Dan-Shen (DanS)	丹參	→	Jia-Wei-Xiao-Yao-San (JWXYS)	加味逍遙散	124	3.28	28.12	2.18
Da-Huang (DaH)	大黃	→	Yin-Chen-Wu-Ling-San (YCWLS)	茵陳五苓散	114	3.01	31.75	2.23
Zhi-Zi (ZZ)	梔子	→	Da-Huang (DaH)	大黃	112	2.96	56.85	5.99
Long-Dan-Xie-Gan-Tang (LDXGT)	龍膽瀉肝湯	→	Jia-Wei-Xiao-Yao-San (JWXYS)	加味逍遙散	103	2.72	30.38	2.36
Yin-Chen-Hao-Tang (YCHT)	茵陳蒿湯	→	Jia-Wei-Xiao-Yao-San (JWXYS)	加味逍遙散	100	2.64	30.03	2.33
Ban-Xia-Xie-Xin-Tang (BXXXT)	半夏瀉心湯	→	Yin-Chen-Hao-Tang (YCHT)	茵陳蒿湯	99	2.62	42.86	4.87
San-Qi (SanQ)	三七	→	Jia-Wei-Xiao-Yao-San (JWXYS)	加味逍遙散	96	2.54	46.83	3.64
Zhi-Zi (ZZ)	梔子	→	Bai-Hua-She-She-Cao (BHSSC)	白花蛇舌草	79	2.09	40.10	7.48
Bai-Hua-She-She-Cao (BHSSC)	白花蛇舌草	→	Yin-Chen-Wu-Ling-San (YCWLS)	茵陳五苓散	76	2.01	37.44	2.62
Da-Huang (DaH)	大黃	→	Dan-Shen (DanS)	丹參	63	1.66	17.55	1.51
San-Qi (SanQ)	三七	→	Dan-Shen (DanS)	丹參	65	1.72	31.71	2.72
San-Qi (SanQ)	三七	→	Long-Dan-Xie-Gan-Tang (LDXGT)	龍膽瀉肝湯	65	1.72	31.71	3.54
San-Qi (SanQ)	三七	→	Ban-Xia-Xie-Xin-Tang (BXXXT)	半夏瀉心湯	52	1.37	25.37	4.16
San-Qi (SanQ)	三七	→	Yin-Chen-Hao-Tang (YCHT)	茵陳蒿湯	63	1.66	30.73	3.49
Ban-Xia-Xie-Xin-Tang (BXXXT)	半夏瀉心湯	→	Jia-Wei-Xiao-Yao-San (JWXYS)	加味逍遙散	83	2.19	35.93	2.79
Ban-Xia-Xie-Xin-Tang (BXXXT)	半夏瀉心湯	→	Long-Dan-Xie-Gan-Tang (LDXGT)	龍膽瀉肝湯	94	2.48	40.69	4.54

*CHM* Chinese herbal medicine; *LHS* left-hand-side; *RHS* right-hand-side

Support (X) (%) = Frequency of prescriptions of X and Y products/total prescriptions × 100%

Confidence (X → Y) (%) = Frequency of prescriptions of X and Y products / Frequency of prescriptions of X product × 100%

P (Y) (%) = Frequency of prescriptions of Y product / total prescriptions × 100%

Lift = Confidence (X → Y) (%) / P (Y) (%)

## Results

The flow chart for the identification of patients with decompensated liver cirrhosis is presented in Fig. 1. A total of 2467 patients with decompensated liver cirrhosis were identified for the period between the years 2000 and 2009 from the registry for patients with catastrophic illness in Taiwan. After exclusion of patients based on the exclusion criteria, a total of 726 patients with decompensated liver cirrhosis were included in the analysis. Of these, 149 were in the CHM users’ group, while 577 were in the CHM non-users’ group. Differences were found in gender and income between these 2 groups (*p* < 0.05; Table 1), with more females and higher income in the CHM group. After matching for age, gender, and Charlson comorbidity index score, 149 and 298 patients were included in the CHM user and CHM non-user groups, respectively (Fig. 1 and Table 1). The only difference observed between these two matched groups was higher income in the CHM group (*p* < 0.05; Table 1).

As shown in Table 2, CHM users had a lower overall mortality risk compared with CHM non-users (Crude: *p* < 0.0001; HR: 0.54, 95% CI: 0.43–0.68). After adjusting for age, gender, Charlson comorbidity index score, and income; CHM users still had a lower overall mortality risk than those CHM non-users (Multivariable: *p* < 0.0001; HR: 0.54, 95% CI: 0.42–0.69). Parameters for counting the cumulative incidence of overall mortality for CHM users and CHM non-users are shown in Table S7 and Table S8. Kaplan-Meier survival curves showed that the cumulative incidence of overall mortality in CHM users was lower than that in CHM non-users (stratified log-rank test, *p* = 0.0002; Fig. 2). The average survival time for CHM users was 4.232 years, while the average survival time for CHM non-users was 2.569 years (Table S6).

The commonly used Chinese herbal formulas and single herbs and their compositions used for the treatment of patients with decompensated liver cirrhosis are listed in Table S1. Results of the association rules and network analysis, i.e., the support (%), confidence (%), and lift of the association rules of these most commonly used co-prescription pairs are shown in Table 3 and Fig. 3. The strongest CHM co-prescription pattern (Yin-Chen-Hao-Tang (YCHT) → Long-Dan-Xie-Gan-Tang (LDXGT); support: 3.81%, confidence: 43.24%, lift: 4.83) showed the highest value of support data, which suggests that this co-prescription pattern had the most significant association for the treatment of decompensated liver cirrhosis. This was followed by Zhi-Zi (ZZ) → Yin-Chen-Wu-Ling-San (YCWLS) (support: 3.57%, confidence: 68.53%, lift: 4.80) and Bai-Hua-She-She-Cao (BHSSC) → Da-Huang (DaH) (support: 3.38%, confidence: 63.05%, lift: 6.65).

To explore the CHM network, the CHM combinations, co-prescription patterns and constituted networks were identified and drawn (Fig. 3). There was one main CHM cluster with Yin-Chen-Hao-Tang (YCHT), Long-Dan-Xie-Gan-Tang (LDXGT), Ban-Xia-Xie-Xin-Tang (BXXXT), Jia-Wei-Xiao-Yao-San (JWXYS), Dan-Shen (DanS), and San-Qi (SanQ). The second main CHM cluster was Yin-Chen-Wu-Ling-San (YCWLS), Bai-Hua-She-She-Cao (BHSSC), Da-Huang (DaH), and Zhi-Zi (ZZ). We observed that the use of CHM as adjunct therapy may reduce the risk of overall mortality in decompensated liver cirrhosis patients. These CHM co-prescription patterns may exhibit anti-fibrotic effects in the liver and may show protective effects against overall mortality.

## Discussion

This study has shown that CHM treatment is associated with a lower overall mortality risk in patients with decompensated liver cirrhosis. Among these patients, the strongest CHM co-prescription pattern (YCHT → LDXGT) had the highest value of support data, followed by ZZ → YCWLS and BHSSC → DaH. The study was aimed at exploring the potential effects of these herbs on liver fibrosis and the subsequent overall mortality in these patients.

Liver cirrhosis is a late stage of scarring in the liver characterized by a complex consisting of different kinds of extracellular matrix (ECM) proteins such as type I, III, and IV collagen proteins, elastic fibers, fibronectin, laminin, and proteoglycans [19].. Alcoholism, hepatitis B and/or hepatitis C virus infections, and nonalcoholic steatohepatitis may induce a wound healing response (fibrogenesis), and extra synthesis of ECM with an overexpression of tissue inhibitors of matrix metalloproteinases (TIMPs) [20]. Currently, there are several kinds of anti-fibrotic drug candidates that have shown anti-fibrotic activities in vitro, in animal studies, and/or in clinical patients [21, 22]. These include cytokine antagonists, phosphodiesterase inhibitors, matrix metalloproteinase (MMP) inducers, prostanoids, vasoactive modulators, histone deacetylase inhibitors, peroxisome proliferator-activated receptor (PPAR)-alpha agonists, PPAR-gamma agonists, plant-derived drugs, and farnesoid-X-receptor agonists [4, 21]. The drug candidates can be used in combination, either for long-term or for short-term; however, the long-term safety of the combination of these anti-fibrotic drug candidates for liver cirrhosis patients remains to be elucidated.

The results of this study suggest that CHM may have protective effect in these patients with decompensated liver cirrhosis. Similar results were also observed in the total subjects (before matching) (Table 1, Table S2, and Fig. S1). These results suggested that CHM treatment was associated with a lower overall mortality risk in decompensated liver cirrhosis patients in both of the total and matched subjects. Furthermore, CHM users had a lower overall mortality risk than CHM non-users from both of the database of the registry for catastrophic illness patients and the database of outpatient and inpatient, suggesting that there may not be a selection bias in our study (Table S3, Table S4, Fig, S2, and Fig. S3). Truly, there is increasing evidence that CHM has long been used for the clinical treatment of liver diseases in Taiwan [15, 23–26]. Our results provide a motivation to investigate these CHM for pharmacological effects in decompensated liver cirrhosis. Among the co-prescription patterns determined by using the association rule mining for decompensated liver cirrhosis patients, the strongest CHM co-prescription pattern YCHT → LDXGT resulted in the highest support, followed by ZZ → YCWLS and BHSSC → DaH. These three stronger co-prescription patterns seem to be independent of each other according to the CHM network analysis. Based on the theory of TCM, YCHT and LDXGT are strong CHM prescriptions used to eliminate heat and dampness from the human body and to improve liver regulation and jaundice remission. Indeed, they have been used in treating liver diseases in ancient China and are still used to treat chronic hepatitis in Taiwan [27, 28]. YCHT comprises three single herbs - Yin-Chen-Hao (YCH; *Artemisia capillaris Thunb.*), ZZ (*Gardenia jasminoides J.Ellis*), and DaH (*Rheum palmatum L.*). YCWLS is a herbal formula derived from YCHT and is frequently prescribed for chronic hepatitis and liver cirrhosis in Taiwan [15, 27]. YCHT has been shown to protect the liver from fibrosis and oxidative stress in rat or mouse models [29–34]. Among YCHT, herbal extracts from YCH; *Artemisia capillaris Thunb.* and DaH; *Rheum palmatum L.* have shown anti-fibrotic effects in rat livers [35, 36]. Chlorogenic acid and umbelliferone are the natural compounds of YCH and protect from liver fibrosis [37–39]. Geniposide, genipin, and crocin are the natural compounds of ZZ and also exhibit anti-fibrotic activity in the liver [40–42]. Emodin and rhein are the natural compounds of DaH and alleviate liver fibrosis [43–45]. LDXGT is composed of ten single herbs [27]. Three natural compounds - swertiamarin, geniposide, and baicalin have been identified in LDXGT and have shown anti-fibrotic activity in the liver [40, 46–48]. BHSSC; *Oldenlandia diffusa (Willd.) Roxb.* is well-known for the treatment of hepatitis and liver cancer [49, 50]. Oleanolic acid and ursolic acid are two natural compounds of BHSSC [51]. A derivative of oleanolic acid has shown anti-fibrotic effects in rat livers [52]. Ursolic acid has shown to exhibit anti-fibrotic effects in the liver of mice and rats [53–56].

Our co-prescription patterns and network analysis have shown that YCHT, YCWLS, LDXGT, and BHSSC exhibited anti-fibrotic activity in the liver and may attenuate overall mortality in decompensated liver cirrhosis. This study has shown that adjunct therapy using CHM may be useful for attenuating overall mortality in patients with decompensated liver cirrhosis.

The limitations of this study include the lack of information about patient education, lifestyle-diet, behavior, occupation, and blood biochemical analysis of liver function and other clinical diagnostic data. This study provides co-prescription patterns with potential protective effects in these patients, which can show a direction for future investigations regarding the safety and efficacy of these agents against liver fibrosis, and possible drug interactions. Further, prospective studies or ad hoc designed clinical trials are necessary in this regard. Functional investigation of CHM and related natural compounds protective effects against liver fibrosis are also necessary.

## Conclusions

CHM usage exhibited a lower hazard ratio for the risk of overall mortality. The strongest CHM co-prescription pattern YCHT → LDXGT caused the highest support, followed by ZZ → YCWLS and BHSSC → DaH. The use of CHM may reduce the risk of overall mortality in patients with decompensated liver cirrhosis; however, further studies are required to optimize the safety and efficacy.

## Supplementary information

**Additional file 1 Figure S1.** The cumulative incidence of overall mortality in patients with decompensated liver cirrhosis based on Chinese herbal medicine (CHM) usage (before matching). **Figure S2.** A flow diagram presenting the study participants enrollment (from the database of outpatient and inpatient). **Figure S3.** The cumulative incidence of overall mortality in patients with decompensated liver cirrhosis based on Chinese herbal medicine (CHM) usage (from the database of outpatient and inpatient). **Table S1** Composition of the most commonly used herbal formulas and single herbs in patients with decompensated liver cirrhosis in Taiwan. **Table S2** Hazard ratios (95% CI) for overall mortality of patients with decompensated liver cirrhosis (before matching). **Table S3** Demographic characteristics of patients with decompensated liver cirrhosis according to CHM usage in Taiwan (from the database of outpatient and inpatient). **Table S4** Hazard ratios (95% CI) for overall mortality of patients with decompensated liver cirrhosis (from the database of outpatient and inpatient). **Table S5.** Distribution of the cumulative period of CHM treatment of CHM users of patients with decompensated liver cirrhosis in this study in Taiwan (during study period after the index date). **Table S6.** Average survival time of patients with decompensated liver cirrhosis between CHM and CHM non-users. **Table S7.** Parameters for counting the cumulative incidence of overall mortality at Fig. 2 for CHM non-users. **Table S8.** Parameters for counting the cumulative incidence of overall mortality at Fig. 2 for CHM users.

## Data Availability

The data that support the findings of this study are available from the National Health Research Institute (NHRI), but restrictions apply to the availability of these data, which were used under license for the current study, and so are not publicly available.

## Abbreviations

HCC: Hepatocellular carcinoma
CAM: Complementary and alternative medicine
CHM: Chinese herbal medicine
NHIRD: National Health Insurance Research database
HR: Hazard ratio
ICD-9-CM: international Classification of Disease, 9th Revision, Clinical Modification
YCHT: Yin-Chen-Hao-Tang
LDXGT: Long-Dan-Xie-Gan-Tang
ZZ: Zhi-Zi
YCWLS: Yin-Chen-Wu-Ling-San
BHSSC: Bai-Hua-She-She-Cao
DaH: Da-Huang
