# Supplementary material for: Decreased overall mortality rate with Chinese herbal medicine usage in patients with decompensated liver cirrhosis in Taiwan
Source: BMC Complement Med Ther. 2020 Jul 14;20:221. doi: 10.1186/s12906-020-03010-6 (PMC7362535; doi:10.1186/s12906-020-03010-6)
Supplement: Supplementary file 1 — Additional file 1 Figure S1. The cumulative incidence of overall mortality in patients with decompensated liver cirrhosis based on Chinese herbal medicine (CHM) usage (before matching). Figure S2. A flow diagram presenting the study participants enrollment (from the database of outpatient and inpatient). Figure S3. The cumulative incidence of overall mortality in patients with decompensated liver cirrhosis based on Chinese herbal medicine (CHM) usage (from the database of outpatient and inpatient). Table S1 Composition of the most commonly used herbal formulas and single herbs in patients with decompensated liver cirrhosis in Taiwan. Table S2 Hazard ratios (95% CI) for overall mortality of patients with decompensated liver cirrhosis (before matching). Table S3 Demographic characteristics of patients with decompensated liver cirrhosis according to CHM usage in Taiwan (from the database of outpatient and inpatient). Table S4 Hazard ratios (95% CI) for overall mortality of patients with decompensated liver cirrhosis (from the database of outpatient and inpatient). Table S5. Distribution of the cumulative period of CHM treatment of CHM users of patients with decompensated liver cirrhosis in this study in Taiwan (during study period after the index date). Table S6. Average survival time of patients with decompensated liver cirrhosis between CHM and CHM non-users. Table S7. Parameters for counting the cumulative incidence of overall mortality at Fig. 2 for CHM non-users. Table S8. Parameters for counting the cumulative incidence of overall mortality at Fig. 2 for CHM users. [file 12906_2020_3010_MOESM1_ESM.docx]

**Supplementary Information**

Supplementary Text

Figures S1-S3

Tables S1-S8

**Supplementary Text**

**Figure S1.** The cumulative incidence of overall mortality in patients with decompensated liver cirrhosis based on Chinese herbal medicine (CHM) usage (before matching).

**Figure S2.** A flow diagram presenting the study participants enrollment (from the database of outpatient and inpatient).

**Figure S3.** The cumulative incidence of overall mortality in patients with decompensated liver cirrhosis based on Chinese herbal medicine (CHM) usage (from the database of outpatient and inpatient).

**Table S1** Composition of the most commonly used herbal formulas and single herbs in patients with decompensated liver cirrhosis in Taiwan.

**Table S2** Hazard ratios (95% CI) for overall mortality of patients with decompensated liver cirrhosis (before matching).

**Table S3** Demographic characteristics of patients with decompensated liver cirrhosis according to CHM usage in Taiwan (from the database of outpatient and inpatient).

**Table S4** Hazard ratios (95% CI) for overall mortality of patients with decompensated liver cirrhosis (from the database of outpatient and inpatient).

**Table S5.** Distribution of the cumulative period of CHM treatment of CHM users of patients with decompensated liver cirrhosis in this study in Taiwan (during study period after the index date).

**Table S6.** Average survival time of patients with decompensated liver cirrhosis between CHM and CHM non-users.

**Table S7.** Parameters for counting the cumulative incidence of overall mortality at figure 2 for CHM non-users.

**Table S8.** Parameters for counting the cumulative incidence of overall mortality at figure 2 for CHM users.

Fig. S1


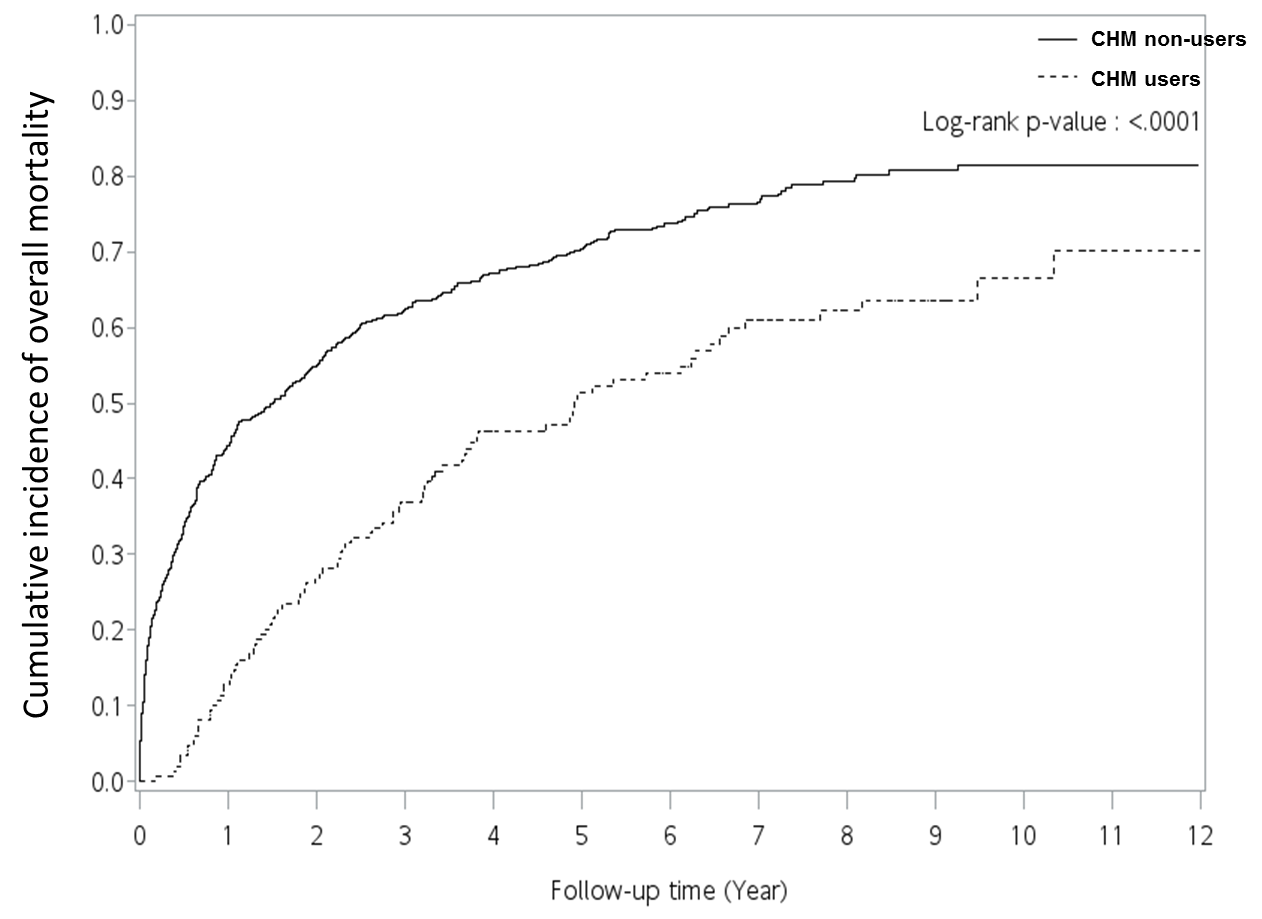


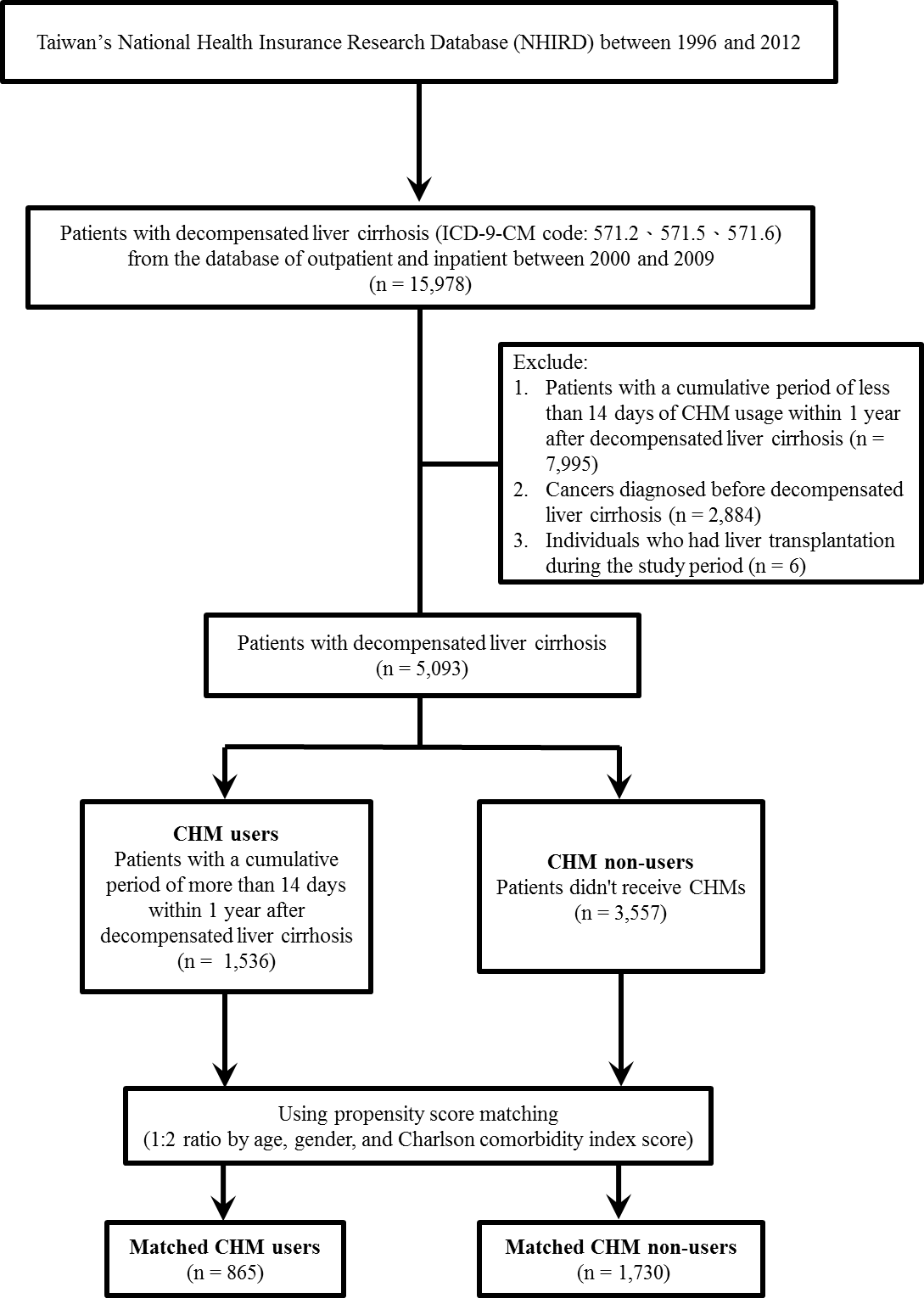
Fig. S2

Fig. S3


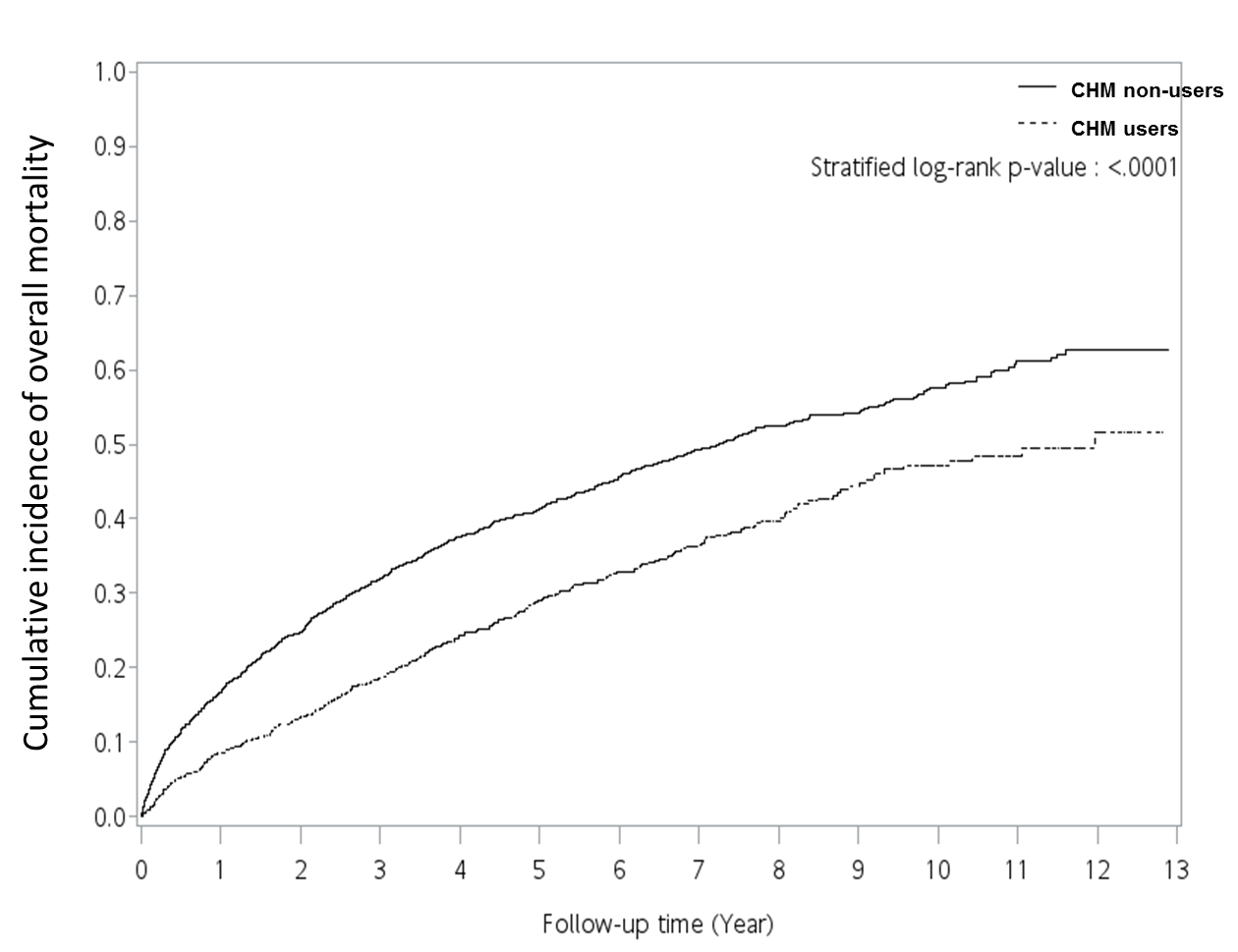


| **Table S1** Composition of the most commonly used herbal formulas and single herbs in decompensated liver cirrhosis patients in Taiwan | | | | | | | | |
| --- | --- | --- | --- | --- | --- | --- | --- | --- |
| **Formulas** | **Chinese name** | **Number of herbs** | **Composition (Pin-yin name (latin name; botanical plant name))** | **Frequency of prescriptions** | **Person-year** | **Frequency of user (N (%))** | **Average drug dose per day (g)** | **Average duration for prescription (days)** |
| **Total** |  |  |  | **3784** | **631.1** | **149 (100.0%)** | **11.8** | **8.6** |
| **Herbal formula (Pin-yin name)** |  |  |  | **3695** | **630.6** | **148 (99.3%)** | **8.5** | **8.6** |
| Yin-Chen-Wu-Ling-San (YCWLS) | 茵陳五苓散 | 6 | **Yin-Chen-Hao** (*Artemisia capillaris*; *Artemisia capillaris Thunb.*), **Ze-Xie** (*Alisma Plantago-Aquatica*; *Alisma plantago-aquatica L.*), **Fu-Ling** (*Wolfiporia extensa*; *Wolfiporia extensa (Peck) Ginns*), **Zhu-Ling** (*Polyporus umbellatus*; *Polyporus umbellatus (Pers.) Fr.*), **Bai-Zhu** (*Atractylodes Macrocephala*; *Atractylodes macrocephala Koidz.*), **Gui-Zhi** (*Cinnamomum cassia*; *Cinnamomum cassia (L.) J.Presl*) | 540 | 249.6 | 52 (34.9%) | 2.9 | 9.1 |
| Jia-Wei-Xiao-Yao-San (JWXYS) | 加味逍遙散 | 10 | **Dang-Gui** (*Angelica sinensis*; *Angelica sinensis (Oliv.) Diels*), **Bai-Shao** (*Paeonia lactiflora*; *Paeonia lactiflora Pall.*), **Fu-Ling** (*Wolfiporia extensa*; *Wolfiporia extensa (Peck) Ginns*), **Bai-Zhu** (*Atractylodes Macrocephala*; *Atractylodes macrocephala Koidz.*), **Chai-Hu** (*Bupleurum falcatum*; *Bupleurum falcatum L.*), **Mu-Dan-Pi** (*Paeonia officinalis*; *Paeonia officinalis L.*), **Zhi-Zi** (*Gardenia jasminoides*; *Gardenia jasminoides J.Ellis*), **Gan-Cao** (*Glycyrrhiza uralensis*; *Glycyrrhiza uralensis Fisch.*), **Bo-He** (*Mentha arvensis*; *Mentha arvensis L.*), **Sheng-Jiang** (*Zingiber officinale*; *Zingiber officinale Roscoe*) | 488 | 278.4 | 55 (36.9%) | 3.9 | 9.8 |
| Long-Dan-Xie-Gan-Tang (LDXGT) | 龍膽瀉肝湯 | 10 | **Long-Dan-Cao** (*Gentiana scabra*; *Gentiana scabra Bunge*), **Huang-Qin** (*Scutellaria baicalensis*; *Scutellaria baicalensis Georgi*), **Zhi-Zi** (*Gardenia jasminoides*; *Gardenia jasminoides J.Ellis*), **Mu-Tong** (*Akebia quinata*; *Akebia quinata (Houtt.) Decne.*), **Che-Qian-Zi** (*Plantago depressa*; *Plantago depressa Willd.*), **Ze-Xie** (*Alisma Plantago-Aquatica*; *Alisma plantago-aquatica L.*)*,***Chai-Hu** (*Bupleurum falcatum*; *Bupleurum falcatum L.*),**Sheng-Di-Huang** (*Rehmannia glutinosa*; *Rehmannia glutinosa (Gaertn.) DC.*), **Dang-Gui** (*Angelica sinensis*; *Angelica sinensis (Oliv.) Diels*), **Gan-Cao** (*Glycyrrhiza uralensis*; *Glycyrrhiza uralensis Fisch.*) | 341 | 209.2 | 38 (25.5%) | 2.2 | 8 |
| Yin-Chen-Hao-Tang (YCHT) | 茵陳蒿湯 | 3 | **Yin-Chen-Hao** (*Artemisia capillaris*; *Artemisia capillaris Thunb.*), **Zhi-Zi** (*Gardenia jasminoides*; *Gardenia jasminoides J.Ellis*), **Da-Huang** (*Rheum palmatum*; *Rheum palmatum L.*) | 334 | 118.8 | 27 (18.1%) | 2.5 | 8.8 |
| Ban-Xia-Xie-Xin-Tang (BXXXT) | 半夏瀉心湯 | 7 | **Zhi-Ban-Xia** (*Pinellia ternata*; *Pinellia ternata (Thunb.) Makino*), **Gan-Jiang** (*Zingiber officinale*; *Zingiber officinale Roscoe*), **Huang-Qin** (*Scutellaria baicalensis*; *Scutellaria baicalensis Georgi*), **Huang-Lian** (*Coptis chinensis*; *Coptis chinensis Franch.*), **Ren-Shen** ( *Panax ginseng*; *Panax ginseng C.A.Mey.*), **Da-Zao** (*Ziziphus jujuba*; *Ziziphus jujuba Mill.*), **Zhi-Gan-Cao** (*Glycyrrhiza uralensis*; *Glycyrrhiza uralensis Fisch.)* | 231 | 117.9 | 26 (17.4%) | 2.4 | 7.1 |
| **Single herbs (Pin-yin name)** |  |  |  | **3194** | **597.2** | **140 (94.0%)** | **4.1** | **8.9** |
| Dan-Shen (DanS) | 丹參 | 1 | **Dan-Shen** (*Salvia miltiorrhiza*; *Salvia miltiorrhiza Bunge*) | 443 | 269.7 | 53 (35.6%) | 1.1 | 12.2 |
| Da-Huang (DaH) | 大黃 | 1 | **Da-Huang** (*Rheum palmatum*; *Rheum palmatum L.*) | 359 | 113.7 | 23 (15.4%) | 0.5 | 10.8 |
| San-Qi (SanQ) | 三七 | 1 | **San-Qi** (*Panax notoginseng*; *Panax notoginseng (Burkill) F.H.Chen*) | 205 | 138.9 | 30 (20.1%) | 1 | 9.3 |
| Bai-Hua-She-She-Cao (BHSSC) | 白花蛇舌草 | 1 | **Bai-Hua-She-She-Cao** (*Oldenlandia diffusa*; *Oldenlandia diffusa (Willd.) Roxb.*) | 203 | 47.7 | 12 (8.1%) | 1.2 | 10.9 |
| Zhi-Zi (ZZ) | 梔子 | 1 | **Zhi-Zi** (*Gardenia jasminoides*; *Gardenia jasminoides J.Ellis*) | 197 | 100.3 | 18 (12.1%) | 1.2 | 8.9 |
| *Sorted by frequency of prescriptions. | | | | | | | | |
| Information is obtained from the websites (http://www.americandragon.com/index.htm; http://old.tcmwiki.com/; http://www.shen-nong.com/eng/front/index.html; http://www.ipni.org/; http://www.theplantlist.org/). | | | | | | | | |

| **Table S2** Hazard ratios (95% CI) for overall mortality of patients with decompensated liver cirrhosis (before matching) | | | | | | | | |
| --- | --- | --- | --- | --- | --- | --- | --- | --- |
|  | **Number of death (n = 524)** | **Total (n = 726)** | **Crude** | | | **Multivariable** | | |
|  | **N (%)** | **N** | **Hazard ratio** | **95% CI** | ***p*-value** | **Hazard ratio** | **95% CI** | ***p*-value** |
| **CHM use (vs. non-CHM use)** |  |  |  |  |  |  |  |  |
| No | 436 (75.56%) | 577 | Ref. | Ref. | Ref. | Ref. | Ref. | Ref. |
| Yes | 88 (59.06%) | 149 | 0.53 | (0.42-0.66) | ***<0.0001*** | 0.55 | (0.43-0.69) | ***<0.0001*** |
| **Age (per year)** | ND | ND | 1.01 | (1.01-1.02) | ***<0.0001*** | 1.01 | (1.00-1.02) | ***0.0221*** |
| **Gender** |  |  |  |  |  |  |  |  |
| Male | 397 (70.52%) | 563 | Ref. | Ref. | Ref. | Ref. | Ref. | Ref. |
| Female | 127 (77.91%) | 163 | 1.24 | (1.02-1.52) | ***0.0348*** | 1.13 | (0.90-1.41) | 0.2903 |
| **Charlson comorbidity index score (per score)** | ND | ND | 1.07 | (1.03-1.11) | ***0.0003*** | 1.04 | (1.01-1.08) | ***0.0204*** |
| **Income** |  |  |  |  |  |  |  |  |
| <NT20,000 | 365 (77.99%) | 468 | Ref. | Ref. | Ref. | Ref. | Ref. | Ref. |
| NT20,000-NT30,000 | 102 (60.71%) | 168 | 0.64 | (0.51-0.79) | ***<0.0001*** | 0.67 | (0.54-0.83) | ***0.0003*** |
| ≧NT30,000 | 57 (63.33%) | 90 | 0.65 | (0.49-0.86) | ***0.0026*** | 0.74 | (0.56-0.98) | ***0.0361*** |
| CHM, Chinese herbal medicine; HR, hazard ratio; 95% CI, 95% confidence interval; Ref., reference; ND, not determined. | | | | | | | | |
| Adjusted factors included age, gender, Charlson comorbidity index score, CHM use, and income. | | | | | | | | |
| Cox proportional hazard models were applied in this analysis. | | | | | | | | |

| **Table S3** Demographic characteristics of patients with decompensated liver cirrhosis according to CHM usage in Taiwan (from the database of outpatient and inpatient) | | | | | | |
| --- | --- | --- | --- | --- | --- | --- |
| **Characteristics** | **Total subjects** | | ***p*-value** | **Matched subjects** | | ***p*-value** |
|  | **CHM users** | **Non-CHM users** |  | **CHM users** | **Non-CHM users** |  |
|  | **(N=1,536)** | **(N=3,557)** |  | **(N=865)** | **(N=1,730)** |  |
|  | **N (%)** | **N (%)** |  | **N (%)** | **N (%)** |  |
| **Age (Mean±SD)** | 55.9± 13.7 | 59.25± 16.23 | ***<0.001*** | 55.72± 14.12 | 55.52± 15.53 | 0.744 |
| **Gender** |  |  | ***<0.001*** |  |  | 0.081 |
| Male | 978 (63.67%) | 2750 (77.31%) |  | 696 (80.46%) | 1440 (83.24%) |  |
| Female | 558 (36.33%) | 807 (22.69%) |  | 169 (19.54%) | 290 (16.76%) |  |
| **Charlson comorbidity index score (Mean±SD)** | 2.82± 1.9 | 3.33± 2.4 | ***<0.001*** | 2.76± 1.85 | 2.64± 1.85 | 0.124 |
| **Interferon therapy** | 37 (2.41%) | 30 (0.84%) | ***<0.001*** | 15 (1.73%) | 14 (0.81%) | ***0.035*** |
| **Anti-virus therapy** | 36 (2.34%) | 29 (0.82%) | ***<0.001*** | 15 (1.73%) | 14 (0.81%) | ***0.035*** |
| **Income** |  |  | ***<0.001*** |  |  | ***<0.001*** |
| <NT20,000 | 739 (48.11%) | 2274 (63.93%) |  | 431 (49.83%) | 1024 (59.19%) |  |
| NT20,000-NT30,000 | 470 (30.60%) | 818 (23.00%) |  | 243 (28.09%) | 432 (24.97%) |  |
| ≧NT30,000 | 327 (21.29%) | 465 (13.07%) |  | 191 (22.08%) | 274 (15.84%) |  |
| **Urbanization level** |  |  | ***<0.001*** |  |  | ***0.007*** |
| 1 | 650 (45.49%) | 1777 (53.12%) |  | 366 (45.58%) | 854 (52.33%) |  |
| 2 | 426 (29.81%) | 869 (25.98%) |  | 241 (30.01%) | 425 (26.04%) |  |
| 3 | 353 (24.7%) | 699 (20.9%) |  | 196 (24.41%) | 353 (21.63%) |  |
| *p*-values for gender, interferon therapy, anti-virus therapy, income, and urbanization level were calculated with chi-square test. | | | | | | |
| For matched subjects, *p*-values for age and Charlson comorbidity index score were calculated using paired Student’s t-test. | | | | | | |
| CHM, Chinese herbal medicine; N, number. | | | | | | |
| These comorbidities were identified before decompensated liver cirrhosis. | | | | | | |
| Propensity score matching was performed for CHM and non-CHM users in 1:2 ratio for age, sex, and Charlson comorbidity index score. | | | | | | |
| Urbanization level: 1 indicates the lowest level of urbanization and 3 is the highest level. | | | | | | |

| **Table S4** Hazard ratios (95% CI) for overall mortality of patients with decompensated liver cirrhosis (from the database of outpatient and inpatient) | | | | | | | | |
| --- | --- | --- | --- | --- | --- | --- | --- | --- |
|  | **Number of death (n = 1,082)** | **Total (n = 2,435)** | **Crude** | | | **Multivariable** | | |
|  | **N (%)** | **N** | **Hazard ratio** | **95% CI** | ***p*-value** | **Hazard ratio** | **95% CI** | ***p*-value** |
| **CHM use (vs. non-CHM use)** |  |  |  |  |  |  |  |  |
| No | 789 (48.35%) | 1632 | Ref. | Ref. | Ref. | Ref. | Ref. | Ref. |
| Yes | 293 (36.49%) | 803 | 0.67 | (0.59-0.76) | ***<0.0001*** | 0.69 | (0.60-0.78) | ***<0.0001*** |
| **Age (per year)** | ND | ND | 1.02 | (1.02-1.03) | ***<0.0001*** | 1.01 | (1.01-1.02) | ***<0.0001*** |
| **Gender** |  |  |  |  |  |  |  |  |
| Male | 838 (41.71%) | 2009 | Ref. | Ref. | Ref. | Ref. | Ref. | Ref. |
| Female | 244 (57.28%) | 426 | 1.66 | (1.44-1.90) | ***<0.0001*** | 1.07 | (0.91-1.27) | 0.4280 |
| **Charlson comorbidity index score (per score)** | ND | ND | 1.14 | (1.11-1.18) | ***<0.0001*** | 1.09 | (1.05-1.12) | ***<0.0001*** |
| **Interferon therapy (anti-virus therapy)** |  |  |  |  |  |  |  |  |
| No | 1079 (44.79%) | 2409 | Ref. | Ref. | Ref. | Ref. | Ref. | Ref. |
| Yes | 3 (11.54%) | 26 | 0.24 | (0.08-0.73) | ***0.0114*** | 0.31 | (0.10-0.93) | ***0.0368*** |
| **Income** |  |  |  |  |  |  |  |  |
| <NT20,000 | 730 (54.36%) | 1343 | Ref. | Ref. | Ref. | Ref. | Ref. | Ref. |
| NT20,000-NT30,000 | 230 (35.66%) | 645 | 0.56 | (0.49-0.65) | ***<0.0001*** | 0.61 | (0.52-0.70) | ***<0.0001*** |
| ≧NT30,000 | 122 (27.29%) | 447 | 0.40 | (0.33-0.49) | ***<0.0001*** | 0.49 | (0.41-0.60) | ***<0.0001*** |
| **Urbanization level** |  |  |  |  |  |  |  |  |
| 1 | 555 (45.49%) | 1220 | Ref. | Ref. | Ref. | Ref. | Ref. | Ref. |
| 2 | 284 (42.64%) | 666 | 0.92 | (0.80-1.06) | 0.2386 | 0.98 | (0.85-1.14) | 0.8357 |
| 3 | 243 (44.26%) | 549 | 0.96 | (0.82-1.11) | 0.5587 | 1.00 | (0.86-1.17) | 0.9844 |
| CHM, Chinese herbal medicine; HR, hazard ratio; 95% CI, 95% confidence interval; Ref., reference; ND, not determined. | | | | | | | | |
| Adjusted factors included age, gender, Charlson comorbidity index score, CHM use, interferon therapy (anti-virus therapy), income, and urbanization level. | | | | | | | | |
| Cox proportional hazard models with robust sandwich variance estimator were applied in this analysis. | | | | | | | | |

| **Table S5** Distribution of the cumulative period of CHM treatment of CHM users of patients with decompensated liver cirrhosis in this study in Taiwan (during study period after the index date) | | |
| --- | --- | --- |
| **Cumulative period of CHM treatment (day)** | **CHM users (N = 149)** | |
|  | **N** | **%** |
| **<60 day** | 62 | 41.61 |
| **60-120 day** | 32 | 21.48 |
| **120-240 day** | 21 | 14.09 |
| **>240 day** | 34 | 22.82 |
| N, number; CHM, Chinese herbal medicine. | | |
| Cumulative CHM treatment days was started after the index date during the study period. | | |
| The index date of this study was from the day on which the 14 cumulative days of CHM treatment were completed. | | |

| **Table S6** Average survival time of patients with decompensated liver cirrhosis between CHM and CHM non-users | | | |
| --- | --- | --- | --- |
|  | **Number** | **Average survival time (mean; years)** | **Average survival time (median; years)** |
| **CHM non-users** | **298** | 2.569 | 1.333 |
| **CHM users** | **149** | 4.232 | 3.493 |
| CHM, Chinese herbal medicine. | | | |

| **Table S7** Parameters for counting the cumulative incidence of overall mortality at figure 2 for CHM non-users | | | | | | | | |
| --- | --- | --- | --- | --- | --- | --- | --- | --- |
| **Interval** | | **Effective Sample Size (n)** | **Number of failed (NF)** | **Number of censored (NC)** | **Conditional probability of failure (q)** | **1-conditional probability of failure (1-q)** | **Survival (p)** | **Overall mortality (1-Survival)** |
| **Lower** | **Upper** |  |  |  |  |  |  |  |
| 0 | 1 | 298 | 131 | 0 | 0.4396 | 0.5604 | 1 | 0 |
| 1 | 2 | 167 | 42 | 0 | 0.2515 | 0.7485 | 0.5604 | 0.4396 |
| 2 | 3 | 123.5 | 20 | 3 | 0.1619 | 0.8381 | 0.4195 | 0.5805 |
| 3 | 4 | 93 | 10 | 18 | 0.1075 | 0.8925 | 0.3515 | 0.6485 |
| 4 | 5 | 69.5 | 8 | 9 | 0.1151 | 0.8849 | 0.3137 | 0.6863 |
| 5 | 6 | 51.5 | 5 | 11 | 0.0971 | 0.9029 | 0.2776 | 0.7224 |
| 6 | 7 | 35.5 | 7 | 11 | 0.1972 | 0.8028 | 0.2507 | 0.7493 |
| 7 | 8 | 19.5 | 1 | 7 | 0.0513 | 0.9487 | 0.2012 | 0.7988 |
| 8 | 9 | 14.5 | 1 | 1 | 0.069 | 0.931 | 0.1909 | 0.8091 |
| 9 | 10 | 11 | 0 | 4 | 0 | 1 | 0.1778 | 0.8222 |
| 10 | 11 | 7 | 0 | 4 | 0 | 1 | 0.1778 | 0.8222 |
| Interval (Lower; Upper) means the follow-up time; for example: lower=0; upper=1 means the interval between 0-1 year. | | | | | | | | |
| Effective sample size (n) means the total sample number in the non-CHM users. n = N-1/2 (NC). | | | | | | | | |
| Number of censored (NC) means the number of withdrawal or loss during the interval in the non-CHM users in this study. | | | | | | | | |
| Number of failed (NF) means the number of death during the interval in the non-CHM users in this study. | | | | | | | | |
| Conditional probability of failure (q) = NF/n. | | | | | | | | |
| Survival (p) = ㄇp = ㄇ(1-q). | | | | | | | | |
| Overall mortality = 1-Survival. | | | | | | | | |

| **Table S8** Parameters for counting the cumulative incidence of overall mortality at figure 2 for CHM users | | | | | | | | |
| --- | --- | --- | --- | --- | --- | --- | --- | --- |
| **Interval** | | **Effective Sample Size (n)** | **Number of failed (NF)** | **Number of censored (NC)** | **Conditional probability of failure (q)** | **1-conditional probability of failure (1-q)** | **Survival (p)** | **Overall mortality (1-Survival)** |
| **Lower** | **Upper** |  |  |  |  |  |  |  |
| 0 | 1 | 149 | 28 | 0 | 0.1879 | 0.8121 | 1 | 0 |
| 1 | 2 | 121 | 18 | 0 | 0.1488 | 0.8512 | 0.8121 | 0.1879 |
| 2 | 3 | 101.5 | 14 | 3 | 0.1379 | 0.8621 | 0.6913 | 0.3087 |
| 3 | 4 | 79.5 | 10 | 13 | 0.1258 | 0.8742 | 0.5959 | 0.4041 |
| 4 | 5 | 61.5 | 6 | 3 | 0.0976 | 0.9024 | 0.521 | 0.479 |
| 5 | 6 | 51 | 4 | 6 | 0.0784 | 0.9216 | 0.4701 | 0.5299 |
| 6 | 7 | 40 | 4 | 8 | 0.1 | 0.9 | 0.4333 | 0.5667 |
| 7 | 8 | 29 | 2 | 6 | 0.069 | 0.931 | 0.3899 | 0.6101 |
| 8 | 9 | 19 | 1 | 10 | 0.0526 | 0.9474 | 0.363 | 0.637 |
| 9 | 10 | 11 | 1 | 4 | 0.0909 | 0.9091 | 0.3439 | 0.6561 |
| 10 | 11 | 7 | 0 | 2 | 0 | 1 | 0.3127 | 0.6873 |
| Interval (Lower; Upper) means the follow-up time; for example: lower=0; upper=1 means the interval between 0-1 year. | | | | | | | | |
| Effective sample size (n) means the total sample number in the CHM users. n = N-1/2 (NC). | | | | | | | | |
| Number of censored (NC) means the number of withdrawal or loss during the interval in the CHM users in this study. | | | | | | | | |
| Number of failed (NF) means the number of death during the interval in the CHM users in this study. | | | | | | | | |
| Conditional probability of failure (q) = NF/n. | | | | | | | | |
| Survival (p) = ㄇp = ㄇ(1-q). | | | | | | | | |
| Overall mortality = 1-Survival. | | | | | | | | |
